# Susceptibility of solid organ transplant recipients to viral pathogens with zoonotic potential: A mini-review

**DOI:** 10.1016/j.bjid.2024.103742

**Published:** 2024-04-23

**Authors:** Karine C. Bezerra, Carlos Meton A.G. Vieira, Edmilson F. de Oliveira-Filho, Christian Robson S. Reis, Reinaldo B. Oriá

**Affiliations:** a Universidade Federal do Ceará, Faculdade de Medicina, Laboratório da Biologia da Cicatrização, Ontogenia e Nutrição de Tecidos, Fortaleza, CE, Brazil; b Charité-Universitätsmedizin Berlin, Institute of Virology, Berlin, Germany; c Fundação Oswaldo Cruz, Instituto Aggeu Magalhães, Departamento de Microbiologia, Recife, PE, Brazil

**Keywords:** Zoonosis, Virus, Immunosuppression, Solid organ transplantation, Clinical outcomes, Transplantation failure

## Abstract

A substantial number of zoonotic diseases are caused by viral pathogens, representing a significant menace to public health, particularly to susceptible populations, such as pregnant women, the elderly, and immunocompromised individuals. Individuals who have undergone solid organ transplantation frequently experience immunosuppression, to prevent organ rejection, and, thus are more prone to opportunistic infections. Furthermore, the reactivation of dormant viruses can threaten transplant recipients and organ viability. This mini-review examines the up-to-date literature covering potential zoonotic and organ rejection-relevant viruses in solid organ transplant recipients. A comprehensive list of viruses with zoonotic potential is highlighted and the most important clinical outcomes in patients undergoing transplantation are described. Moreover, this mini-review calls attention to complex multifactorial events predisposing viral coinfections and the need for continuous health surveillance and research to understand better viral pathogens' transmission and pathophysiology dynamics in transplanted individuals.

## Introduction

Infectious diseases are on the rise as an important public health concern due to rapid globalization, intensification of population migratory events, and expansion of livestock trading worldwide. Expanding urban populations, housing overcrowding, poverty, tourism and business traveling, climate change, and environmental predatory exploitation are considered the primary contributing factors of zoonotic-transmitted illnesses in recent years.^1^, ^2^, ^3^ Infectious diseases are highly prevalent in tropical countries with warm and humid climates throughout the year and may be aggravated by adverse environments, inadequate access to healthcare, and poor sanitation and hygiene.

Approximately 75 % of emerging infectious diseases may show a zoonotic transmission potential.^1^ Increased interactions between humans and animals have raised the likelihood of transmitting and spreading zoonotic diseases. Viral pathogens are important zoonotic etiologies, especially to vulnerable populations, encompassing various transmissibility routes and dynamics; most of these may seriously threaten public health.^4^ Virus infections are particularly detrimental to aged and immunocompromised individuals.^5^ Often, the virus infection may be asymptomatic and latent for long years and may arise after acute immune deficiency. One scenario of immunodepression is organ transplantation, as chronic immunotherapy is often required to halt tissue rejection, which may be an end-state adverse effect of graft transplantation. Unfortunately, organ rejection and transplantation failure may occur despite all the healthcare measures taken before the surgical procedure and post-grafting.

In this review, we highlight the main public health-concerned viruses with zoonotic potential and their impact on Solid Organ Transplantation (SOT) outcomes. In addition, we discuss updated findings of possible life-threatening effects of COVID-19 and potential co-infections with other commonly transmitted respiratory viruses, arbovirus, and viruses transmitted through oral-fecal contamination.

### Methodology

A narrative review of the literature was conducted. The literature search utilized three primary biomedical and health databases: PubMed, Scopus, and Science Citation Index (SCI). The search was conducted using the terms (“virus with zoonotic potential” OR “zoonotic virus”) AND (“transplant” OR “transplantation”) AND (“outcome” OR “graft loss” OR “mortality”). The language was not restricted in the search. Excluded from consideration were duplicate publications that did not cover viruses with zoonotic potential, along with scientific meeting summaries. The review was organized to facilitate the reader's understanding according to the following characteristics of zoonoses: epidemiology, taxonomy, clinical manifestations, diagnosis, and other SOT failure-related non-zoonotic pathogens.

### Solid organ transplantation and viral zoonotic infections

SOT is often required to circumvent the health crisis of a life-threatening condition of organ failure due to acute or chronic illnesses or states despite potential major histocompatibility issues. Management of patients subjected to SOT requires a fine health balance between the level of immunosuppression, exposure to opportunistic pathogens, and associated side effects, which may significantly affect prognosis and survival. Opportunistic infections (e.g., driven by dormant viruses in the donor organ or a previous illness from the receptor) may trigger a process of either acute or chronic rejection, a lasting health concern that may be prolonged throughout the patient's entire life. Such a condition justifies a close monitoring of the transplanted organ and the potential side effects of immunosuppressive medications through regular laboratory tests and occasional biopsies. The patient's follow-up for viral and other pathogen coinfections is key for long-term organ transplantation success.

Organ transplantation-associated infections can be classified into three chronological phases that are associated with different etiologies: the initial and immediate postoperative period, typically within the first month, when most of the infections originate from the hospital environment and may be transmitted through the transplanted organ, or are preexisting or driven by postsurgical infections; the second period, from two until six months after surgery, is the period when opportunistic infections thrive; and finally, the third and late phase, beyond six months after transplant surgery, which is characterized by a slight increase in infections, caused by the maintenance of the immunosuppressive state.^6^ Some exceptions are the Herpes Simplex Virus (HSV), which may be reactivated early after transplant, and the varicella-zoster virus, whose clinical manifestation can occur any time after transplantation.^7^

Multiple viral infections may coexist with poor health, and a scenario of coinfection with zoonotic and tissue rejection-related viruses may occur in immunocompromised patients. Cytomegalovirus (CMV) and BK virus are the most prevalent viral infections occurring after kidney transplantation.^5^ Usually, these viruses remain dormant after the initial infection, recurring after immunosuppression and leading to various complications, affecting the lungs, liver, and gastrointestinal tract. Managing and preventing CMV infection is an important aspect of post-transplant care. Before prophylactic protocol was instituted, CMV manifestation occurred during the period of 4 and 6 weeks after transplantation. Currently, with the routine use of antiviral prophylaxis, usually for 3 to 6 months after the transplant, late-onset disease is commonly observed after discontinuation of these drugs.^7^ With a similar mechanism, the use of corticosteroids and calcineurin inhibitors as immunosuppressors compromises the functioning of CD4+ T-lymphocytes, which have an important role in eliminating viruses such as polyomavirus BK, whose opportunistic infection represents a great risk for patients undergoing a kidney transplant, being the cause of viral-induced nephropathy.^8^ Other well-documented viral agents reactivated by immunosuppression, with worldwide distribution in cases of kidney transplants, are herpes simplex virus, varicella-zoster virus, Epstein-Barr virus, hepatitis B virus, and adenovirus, potentially leading to systemic infection or impaired function of the transplanted organ.^5^^,^^7^ Furthermore, specific viruses, such as herpes and polyomavirus, harm the integrity of the host's immune defense, potentiating the risk and effect of other secondary infections. Epstein-Barr virus and human herpesvirus 8 may be the causative agents of lymphoproliferation and cervical cancers after transplant.^9^

**Fig. 1:**
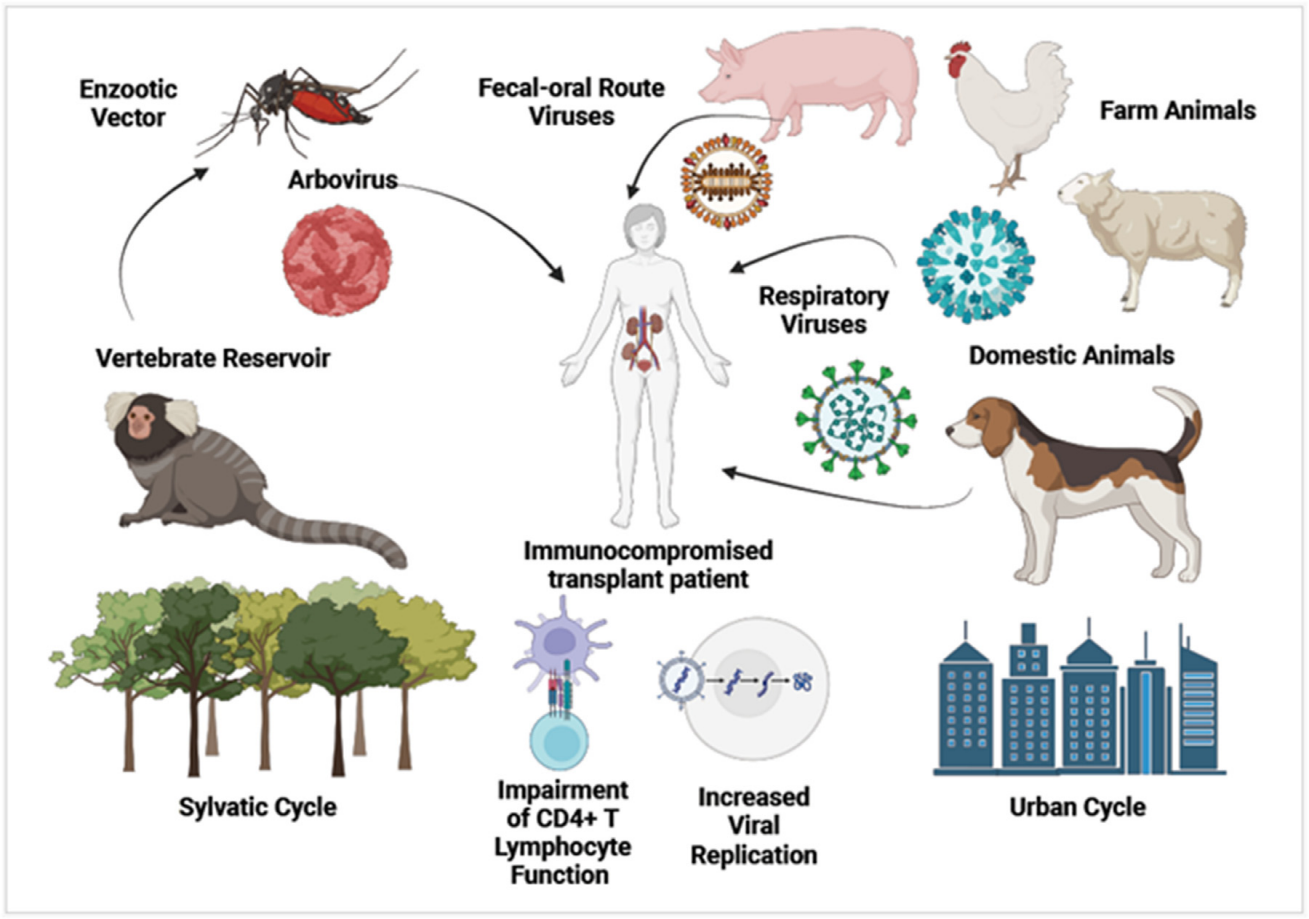
The mechanism diversity of zoonotic viral transmission to immunocompromised transplant recipients, showing the occurrence of intermediate vectors, product consumption of animal origin, and sharing domestic and working environments.

Fig. 1 depicts the diversity of human-animal interactions causing zoonotic viral transmission in immunocompromised transplant patients.

### Potential zoonotic viruses

#### Influenza virus

Influenza viruses belong to the family *Orthomyxoviridae* and comprise an extensive group of pathogenic viruses that cause respiratory diseases, popularly known as flu, being able to transmit between different species of mammals and birds. Transmission occurs through respiratory droplets and can also spread in humans by touching the face with contaminated hands. Typical flu symptoms are fever, chills, cough, sore throat, headaches, muscle or body aches, and fatigue. Vulnerable populations, such as the elderly, pregnant women, and immunosuppressed individuals, can develop more severe diseases, like pneumonia, bronchitis, and exacerbation of chronic medical conditions. Influenza viruses were responsible for several pandemics, with historical records in 1918, 1957, 1968, and 2009, causing many deaths and bringing dramatic public health, social, and economic consequences.

Viruses of the influenza A genus are the most diverse and have zoonotic potential, and the other genres do not demonstrate relevance in interspecies transmission until the present moment. Having an RNA genome, they have two glycoproteins in their envelope, hemagglutinin (HA) and neuraminidase (NA), which are crucial for identifying their serotype. Through the phenomenon of antigenic shift, viruses of the influenza A genus can undergo substantial mutations, potentially causing new epidemic outbreaks. Some varieties are more specific to each host species, while others can occasionally cross over, causing zoonotic transmission to humans.

Recently, a new variety of H5N6 was identified in the Chinese domestic goose, sharing genetic proximity with the virus identified in farmed dogs, suggesting the potential for cross-species transmission. Studies of its genome revealed multiple mutations that can potentially increase affinity with mammalian receptors, increasing its virulence and may represent a public health threat.^10^ The majority of studies investigating the association between SOT and influenza virus infection focused on patients who underwent kidney and lung transplantation, and few studied from heart transplanted individuals, with variable severity of disease but overall decreasing 30-day survival compared to H1N1-infected non-transplanted patients.^11^ A patient underwent liver transplantation after confirmation of having contracted influenza type A by polymerase chain reaction test. Given the urgency of his clinical condition, the team opted to proceed with the transplant while incorporating oral oseltamivir treatment along with postoperative immunosuppression therapy, with a satisfactory recovery of the respiratory condition and liver function.^12^ A retrospective study found, among 84 patients undergoing kidney and liver transplantation with influenza infections, that 65.5% were hospitalized, 16.7% developed pneumonia, and 7.1% were hospitalized in an Intensive Care Unit (ICU). No significant difference in clinically relevant outcomes was observed between kidney and liver-transplanted patients.^13^ A study linked the occurrence of influenza in pediatric recipients of SOT, finding that incidence rates were 2.7% and 7.4% after one and three years, respectively.^14^

#### Hepatitis E virus

Every year, nearly 20 million HEV infections occur, leading to over 3 million symptomatic cases and around 60,000 deaths. In Europe, hepatitis E outbreaks have been observed, with a significant increase in the number of cases, rising from 514 cases in 2005 to 5617 in 2015.^43^ The hepatitis E virus is a single-stranded RNA virus from the *Hepeviridae* family. This small virus presents a pseudo envelope while circulating in the host's bloodstream and a capsid protein that protects the viral RNA. The knowledge of its molecular structure and pathogenicity mechanisms is critical for the future development of vaccines. HEV genotypes 3 and 4 are linked to zoonotic transmission through the fecal-oral route, commonly associated with contaminated water or food. Besides deer and rabbits in forest regions, pigs are often considered the primary reservoir for these genotypes in industrialized regions. Generally, acute HEV infection is either asymptomatic or exhibits mild symptoms, being a self-limited infection, with a mortality rate ranging from 0.5 % to 3 % in young adults. The prodromal phase of acute icteric hepatitis lasts about one week and is characterized by symptoms like fever, myalgia, nausea, and vomiting, followed by the icteric phase,^44^ and most cases do not require specific treatment or management of complications. Hepatitis E can be particularly serious during pregnancy, with a mortality rate of nearly 25 % during the second and third trimesters. Also, for individuals with preexisting liver conditions, it is linked to an increased risk of life-threatening fulminant hepatitis if the acute icteric phase progresses to acute liver failure.

In immunocompromised patients, the infection has the potential to become chronic, especially in individuals undergoing organ transplants or chemotherapy, as well as those with HIV infection,^45^^,^^46^ and in cases of infection by serotypes HEV3 and HEV4, cirrhosis may develop.^44^^,^^47^ In industrialized countries, transmission of the HEV through organ transplantation or blood transfusions has been observed.^46^^,^^47^ A descriptive study investigated the prevalence of positive serology for anti-HEV IgG and the detection of HEV RNA among 192 patients undergoing kidney transplantation, indicating that 23 % of patients had past or current HEV infection.^48^ Another study involved 316 patients also undergoing kidney transplantation, finding a prevalence of 2.5 % of patients with positive anti-HEV IgG serology, and HEV RNA was not detected in any sample studied. Co-infections with HBV and HCV viruses have been observed, including persistent elevations in serum ALT levels.^49^

Chronic HEV infection in patients undergoing SOT is characterized by the persistence of viremia for more than three months after the onset of infection and warrants evaluation for treatment.^50^ In such a scenario, continuously elevated serum aminotransferase levels, evidence of viral activity in organ biopsies, and liver fibrosis have been documented. This scenario is more prevalently associated with the viral serotype HEV3 infection.^51^ With the introduction of a universal HEV RNA screening of deceased organ donors in the UK, early detection and treatment with ribavirin allowed a better prognosis for patients who were at risk of infection after a solid organ transplant.^52^

#### Rabies virus

The rabies virus (RABV) belongs to the family *Rhabdoviridae*, a single-stranded RNA virus. The virus is present in wild and domestic animal reservoirs, and is the etiological agent of a fatal disease, causing 60,000 deaths worldwide every year.^53^ Its transmission most often occurs through the bite of an infected mammal. In 1979, the first report of donor transmission of rabies was through corneal transplantation. After the diagnosis of fatal encephalitis, with rapid neurological deterioration, in four patients receiving kidneys, liver, and a vascular graft from a contaminated donor, there was the first record of RABV transmission through a solid organ transplant.^54^ In 2015, a 22-month-old boy with suspected viral encephalitis died, and having obtained a negative result for serum antibody tests for rabies immunoglobulin, in addition to excluding other pathologies, a kidney and liver transplant was authorized, which caused the death of three patients who received these organs. New-generation sequencing was carried out to obtain the complete genome of the viruses found in the recipients, and phylogenetic analysis indicated great similarity with the RABV lineage circulating in dogs in China.^53^

#### Severe acute respiratory syndrome coronavirus 2 (SARS-CoV-2)

COVID-19 is an infection caused by the SARS-CoV-2, a member of the Coronavirus family, which often induces respiratory distress. Clinical presentation of the disease can be variable, ranging from asymptomatic infected patients to more severe conditions, with some of the symptoms being fever, cough, fatigue, episodes of vomiting, and diarrhea.^55^^,^^56^ In Brazil, there were more than 37 million confirmed cases and approaching 707,000 deaths, with the frequent evaluation of epidemiological data.^57^ The cycle of this disease begins with transmission through the respiratory tract, in which the virus enters the organism from contact with an infected individual. In the initial phase, it replicates rapidly, but the patient may or may not exhibit symptoms. The incubation period occurs from the entry of the pathogen into the body until the presentation of clinical manifestations, lasting from 5 to 7 days.^58^ The diagnosis of COVID-19 can be performed using RT-PCR, which is the gold standard for detecting the SARS-CoV-2 virus.^56^

With the COVID-19 pandemic, more concern has been raised regarding coinfections with zoonotic pathogens, which may escalate to life-threatening and potentially prolonged effects (long COVID-19), especially in more vulnerable populations, including immunosuppressed solid organ transplanted patients.^59^ SARS-CoV-2 has been found in cats,^60^^,^^61^ hamsters,^62^ and deer,^63^^,^^64^ bringing concern about zoonotic events regarding human and animal health.^65^ Despite having human species as a primary reservoir, interspecies contamination of SARS-CoV-2 is a strong possibility through household exposure, close contact between domestic animals and their owners, contamination through contaminated surfaces in the domestic environment, which also raised the possibility of reverse zoonosis, mostly asymptomatic among animals. Another possibility is that animals are reservoirs of pathogens and that through an intermediate vector, they transmit infections indirectly to people. Examining the dynamics of interspecies transmission is crucial when assessing the possibility of reservoir and intermediate hosts that could pose a risk to immunocompromised individuals. It also involves understanding the potential for an ongoing cycle of cross-contamination between species that share proximity.^65^ There is a scarcity of comprehensive data regarding the pathophysiology, transmission levels, and the associated risk to human health from diseases caused by viral transmission in immunocompromised populations, despite the high prevalence of zoonotic events. There is still a gap in knowledge about whether viral coinfections prevail in highly endemic areas of virus circulation during the COVID-19 pandemic and whether these jointly aggravate SARS-CoV-2 infection outcomes.

Considering that the first reported cases of COVID-19 were associated with a seafood market, the concern that it was a zoonosis, although such classification may still be considered premature, preferring the term “emerging infectious disease of probable animal origin”,^66^ and an accumulation of studies has demonstrated a much greater population coverage than exclusively human. SARS-CoV-2 belongs to the SARbecovirus (SARS-related coronavirus), which is a group of coronaviruses massively found in bats. Hence, despite the inability to identify the source of transmission in the initial cases, the zoonotic connection was consistently evident.^67^ Immunocompromised patients use methotrexate, azathioprine, specific monoclonal antibodies, and another immunosuppressive drug. It has been described in the scientific literature that they have greater exposure to the risk of complications, changing the prognosis in the face of a COVID-19 infection, generating great anxiety about their clinical condition, given the possibility of contracting this disease.^68^ There are cases of patients with solid organ transplants infected by the COVID-19 pathology that evolved with severe and unfavorable conditions, mainly due to decreased immunity and greater exposure to the disease virus through organ reception.^69^

#### Arboviral diseases (arthropod-borne viral diseases)

While these diseases are primarily transmitted by arthropods, some of them can have zoonotic components, meaning that they involve animals as reservoir hosts or intermediate hosts, making transmission more complex and expanding the ability to disseminate viral genetic material in the transmission cycle. This group of viruses has shown a significant increase in the number of cases and the emergence of several outbreaks involving Dengue, Chikungunya, and Zika, representing a significant risk for individuals who have undergone organ transplantation. The high levels of viremia among these infections explain the ability to maintain the cycle between the vector and the host.^15^

#### Dengue virus

Dengue virus belongs to the family *Flaviviridae*, transmitted by Aedes mosquitoes, and humans are the primary hosts. Some studies have suggested that non-human primates may also contribute to the transmission cycle, although no reservoirs of the dengue virus have been detected in the Americas.^16^ Given the limited studies of extensive wild areas, this possibility should be considered. Dengue is caused by four related but antigenically distinct dengue viruses covering serotypes 1‒4. Recently, the genome of dengue virus serotype 4 was sequenced in bats' brains. However, this serotype was introduced in Northern Brazil in 2010, an insufficient time to establish an effective sylvatic cycle of the pathogen.^17^ The cycle of this disease begins with the bite of the *Aedes aegypti* mosquito in an infected person. In the mosquito, the virus replicates in its midgut and other organs, up to the salivary glands, migrating to the bloodstream of the bitten individual. There, the virus begins to multiply in organs, such as the spleen and liver; this incubation period lasts about seven days. The dengue virus also can replicate in blood cells, reaching the bone marrow, which compromises the production of platelets. The diagnosis of this disease involves clinical and laboratory aspects, through tests such as serology and agent isolation and determination of specific antibodies, in addition to blood count, ESR (Erythrocyte Sedimentation Rate), coagulogram, and liver enzyme tests.^18^^,^^19^ The authors found that Dengue is relatively infrequent in kidney transplant patients and no disparity in clinical characteristics was observed, compared to immunocompetent patients.^20^ Kidney dysfunction was less severe and transient in kidney transplant recipients.^21^ Only a few kidney transplant recipients had a slight increase in serum creatinine levels without acute renal failure or the need for dialysis support.^22^

On the other hand, the incidence of severe Dengue and mortality were significantly higher in kidney transplant patients.^23^ A study followed four patients diagnosed with dengue infection in their early postoperative period. Among these patients, two required multiple platelet transfusions, one needed intensive care management due to respiratory distress associated with pleural effusion, two patients experienced severe leukopenia, even after interruption of immunosuppressant use, and two presented temporary graft dysfunctions. The authors suggest that screening for Dengue for potential organ transplant candidates and donors is crucial in regions where dengue outbreaks are prevalent. For patients who develop fever and thrombocytopenia shortly after surgery, Dengue NS1 antigen testing should be carried out.^22^

#### West Nile virus

West Nile virus (WNV) is a single-stranded RNA virus, also part of the *Flaviviridae* family, transmitted through mosquito bites and involving a wild cycle that uses birds as hosts. It is widespread worldwide, progressively becoming an important cause of viral encephalitis. Nearly a fifth of infected individuals experience predominantly mild symptoms, while less than 1% of infected persons, especially elderly and immunocompromised individuals, develop severe neuroinvasive disease with possible lasting functional impairment and a 10% mortality risk.^24^

Since 2002, there have been records of WNV transmission in organ recipients, indicating that the risk of neuroinvasive diseases, sequelae, and mortality are significantly higher in the group of transplant patients compared to the general population. However, differently, there are records of asymptomatic cases or cases with full clinical recovery, after contamination with WNV through organ transplantation.^24^ One study involved eight patients receiving SOT, with a reduction in the dosage of immunosuppressants performed in seven patients, and the use of intravenous immunoglobulin in five patients. The electroencephalogram showed abnormality in five patients and persistent neurological dysfunction was recorded in two patients. From this report, two patients died from the viral disease in its neuroinvasive form.^25^

Currently, the most sensitive tests for WNV infection are the detection of IgM antibodies in cerebrospinal fluid and serum enzyme immunoassay. It is important to highlight that samples collected before eight days of the onset of symptoms may provide false negative results, and a new collection must be carried out after this period.^24^

#### Zika virus

Zika is also a component of the *Flaviridae* family and may possess wild reservoirs among non-human primates. The primary transmission mode to humans is through the bite of infected Aedes mosquitoes, primarily Aedes aegypti and Aedes albopictus.^26^ Its transmission can occur non-vectorially through sexual and transplacental transmission.^27^ Most people infected with the Zika virus are asymptomatic, but mild symptoms such as fever, rash, joint and muscle pain, headache, and conjunctivitis may occur, lasting less than a week. A Zika epidemic was recorded on the American continent in 2015 and 2016. This infection reached public prominence due to the occurrence of congenital Zika syndrome, mainly leading to microcephaly, detected in children born to mothers infected during pregnancy.

Case reports describing Zika infection in transplant patients are limited. Zika infection was confirmed among 129 kidney transplants and 58 liver transplants tested in Brazil. All recipients of Zika-infected organ transplant patients experienced complications, mainly bacterial infections, and required hospitalization. Based on this small case series, assessing the potential impact of Zika was not feasible on recipients of immunosuppressed organs, including infectious complications and graft rejection.^28^

A fatal case of Zika infection was described in a patient who underwent a heart transplant eight months earlier, undergoing regular immunosuppressive therapy. The patient developed viral meningitis, which led to the suspension of almost all of these immunosuppressive drugs, which induced an acute rejection and subsequent failure of the transplanted organ.^29^

#### Yellow fever virus

The yellow fever virus belongs to the *Flaviviridae* family, being the cause of a potentially life-threatening disease, frequently transmitted to humans via the bite of infected mosquitoes, mainly Aedes aegypti. In the wild, non-human primates serve as the primary reservoir for the yellow fever virus. Most cases exhibit mild symptoms, such as fever, headache, muscle and joint pain, and fatigue. Unusually, some cases may evolve to a high fever, jaundice, bleeding, and death. The yellow fever vaccine is very effective, achieving long-term immunization, and is recommended as protection for travelers or residents of endemic areas.

Nevertheless, as a live-attenuated vaccine, the transmission of the 17D-yellow fever vaccine virus through SOT was recorded with adverse consequences, demonstrating the need to recognize the possibility of donor-derived infection.^30^ A patient who underwent a kidney transplant five years before, using maintenance immunosuppressants, was inadvertently vaccinated against yellow fever, developing symptoms of the disease and requiring interruption of the medications. One month after the patient was discharged, he developed subclinical antibody-mediated rejection, which was resolved by plasmapheresis.^31^ A patient who had undergone a kidney transplant continued to receive immunosuppressant therapy for 25 years. Due to an outbreak in his area, a vaccination campaign began, but he was not recommended to take the vaccine due to his immunocompromised state. When he developed symptoms of the disease, his health deteriorated, quickly progressing to liver failure, encephalopathy, and death.^32^

#### Chikungunya virus

Chikungunya virus belongs to the Alphavirus genus of the *Togaviridae* family and is transmitted through an arthropod vector. The virus maintains circulation by an urban cycle, transmitted from blood-feeding mosquitoes of the Aedes genus to humans, and a wild cycle, registered only in Africa and Asia, involving mosquitoes and non-human primates as reservoirs. Since 2004, Chikungunya has emerged and has been involved in outbreaks in Africa, Asia, Europe, and the Americas. Recently, the hypothesis that a wild cycle in Mexico involving bats as occasional hosts has emerged.^33^^,^^34^ According to the SINAN (notifiable diseases information system from the Brazilian Ministry of Health), between 2017 and 2021, approximately 784,626 cases of the pathology in question were reported in Brazil.

Regarding regions, the one with the highest number of registered cases was the Brazilian northeast, with an incidence of 415/100 thousand cases.^35^ The specific diagnosis of Chikungunya fever is of paramount importance in endemic regions and is performed through viral isolation and viral RNA research in different samples or through the detection of specific antibodies. Therefore, the standard tests for researching the pathology of interest are serology, RT-PCR, and immunochromatographic tests, among others.^36^ Although Chikungunya fever is generally benign, prolonged polyarthralgia can lead to a significant disability in elderly patients.^37^ Atypical manifestations include meningoencephalitis, myocarditis, and respiratory, renal, and hepatic failure.^38^ A study conducted in Brazil examined the clinical symptoms of Chikungunya in four kidney transplant recipients. The clinical picture was typical; none of the patients developed severe manifestations, and all recovered without complications. Some organ-recipient patients presented with fever, abdominal pain, and headache but did not show arthritis or arthralgia.^39^ A case report details the detection of the virus in the cerebrospinal fluid of a patient undergoing a liver transplant.^40^ A woman who underwent a kidney transplant seven years before was using immunosuppressive drugs when she contracted the infection. The decision was not to change the medication regimen, maintaining the exact dosage. The patient recovered her renal function after convalescence. The immunosuppressants may have lowered the inflammatory response through a blockage of cytokines production, leading to a milder disease and symptoms.^41^

SOT recipients with Chikungunya infection appear to present with a clinical presentation and course similar to what is observed in the general population, with no apparent damage to the graft. Liver transplant recipients did not show an elevation of liver enzymes, and there was no clinical impact on graft function. Although reports of Chikungunya in the transplant population are rare, the transplant community should be reminded that the risk of Chikungunya infection should be considered in deceased organ donor candidates who recently returned from travel to endemic areas. In a case series with ten patients undergoing solid organ transplants, in which five were receptors for a kidney, four were submitted to liver transplant, and one had a combined kidney and liver transplant, only two patients developed arthritis, and none of the study subjects required intensive care.^42^

### Other viruses that may pose a threat through zoonotic transmission

Porcine hemagglutinating encephalomyelitis virus (PHEV) belongs to the *Coronaviridae* family and is known to cause neurological disease in pigs, but it can also affect other animal systems. This virus uses the respiratory route to spread and may cause ataxia, tremors, and inability to stand, having an economic impact on pig farming, with high mortality among piglets. Recently, mutations in the genetic material of this virus were detected, similar to the adaptations presented by human coronavirus, making possible its growth in the respiratory tract.^70^

The Getah virus (GETV) belongs to the *Togaviridae* family and causes a disease that affects primarily horses, pigs, and birds through the bite of mosquitoes. Infrequently, cases of infection in humans have been reported, causing symptoms similar to those of other arboviral diseases, such as fever, joint pain, and rashes. This pathogen may represent an epidemic risk due to its expanding host range and the potential to spread the virus through animal trade, emphasizing the need for vigilance in molecular epidemiology.^71^

Human circoviruses are a group of the *Circoviridae* family; these pathogens have a DNA genome, are small, and do not have an envelope. Most commonly, components of this group infect birds and pigs. However, it has recently been described as human circovirus type 1 (HCirV-1) and was associated with liver damage in a solid organ transplant recipient.^72^

The Lloviu virus (LLOV) belongs to the *Filoviridae* family, including Ebola and Marburg viruses. It has been found in bats, and human infections with the Lloviu virus have been unidentified, but it may infect monkeys and multiple human cell lines, suggesting that the spillover potential of this virus must be a risk to be considered.^73^

Rotavirus has an RNA genome and a triple-layered protein capsid, facilitating its entry into host cells and contributing to its stability and effectiveness in causing gastrointestinal infections in humans and animals, making infection highly contagious. The outer capsid proteins are important for vaccine development and classification of different rotavirus strains. The virus is transmitted via the fecal-oral route, direct contact with an infected person, or the consumption of contaminated food and water. The infection leads to severe diarrhea, vomiting, fever, abdominal pain, and dehydration. A broad genetic diversity of rotaviruses A strains were found among bats, with genetic proximity to contaminating viruses from other mammals, suggesting that they have a common ancestor, opening the possibility that new interspecies contamination may occur.^74^ Rotavirus infection in transplant recipients may precipitate renal failure in severe cases, and enteritis in the context of intestinal transplants demonstrated a 70 % rate of acute cellular rejection.^75^

### Organ transplantation-failure-related viruses

#### BKV polyomavirus

The BK Virus (BKV) is classified within the *Polyomaviridae* family and is a small, non-enveloped virus comprising approximately 5000 nucleotides in length. It is a virus with frequent presence in the human population and a driver of nephropathy and hemorrhagic cystitis in kidney transplanted patients.^76^, ^77^, ^78^ Human polyomaviruses are believed to be transmitted through direct person-to-person contact and via contaminated surfaces, food, and water. However, confirming these transmission routes is difficult due to the asymptomatic nature of primary infections or clinically non-specific presentations.^79^ This pathogen's replication is accelerated in cases of immunosuppression, such as pregnancy, neoplasms, HIV infection, and organ transplantation. There are cases of ureteral stenosis in patients with kidney transplants, which developed after a few months of receiving the organ.^77^ BK virus infection may be a common occurrence at a young age, with the virus remaining dormant in the kidneys of healthy individuals. However, the virus can be reactivated in immunocompromised patients, leading to BK virus-associated nephropathy, characterized by kidney dysfunction and inflammation, increasing the risk of kidney rejection in transplant recipients. Due to this possibility, intensive monitoring and follow-up are required to adjust immunosuppressive therapy during viral reactivation.

#### Cytomegalovirus

The cytomegalovirus (CMV) belongs to the *Herpesviridae* family, having an icosahedral protein capsid and a DNA genome, and unlike other herpesviruses, this pathogen has an external envelope. It has a worldwide prevalence of around 60‒90 % and is associated with poor hygiene, housing, and quality of life conditions. It is rare in immunocompetent children and adults but shows increased morbidity and mortality among immunocompromised individuals.^80^, ^81^, ^82^ Symptoms can range from fever, indisposition, and loss of appetite to cases of jaundice, petechiae, and neurological changes. The absence of antiviral drug prophylaxis may lead to approximately 10 % to 50 % of SOT recipients developing symptomatic CMV infection.^83^ The cycle of this disease initiates with the introduction of the virus into the human body through its attack on the host cell's surface, triggering a replication process and increasing the pathogen burden into blood and other body fluids. Diagnosis of CMV is based on clinical and immunological results by detecting CMV through viral isolation, PCR, IgM, and IgG serology detection by ELISA and western blotting, among other techniques.^81^^,^^84^

Table 1 summarizes representative up-to-date literature on zoonotic viral infection in SOT patients and clinical outcomes.

**Table 1:** Viral pathogens and their clinical presentation in immunocompetent and immunocompromised patients.

Viral pathogen	Clinical presentation		Laboratory diagnosis	References
	Immunocompetent patients	Immunocompromised patients		
Influenza	Fever, chills, cough, sore throat, headaches, muscle or body aches, fatigue	Pneumonia, bronchitis, and exacerbation of chronic medical conditions	Rapid Influenza Diagnostic Tests and Molecular Tests (PCR)	11, 14
Dengue	Fever, headache, joint and muscle pain, rash, nausea, vomiting, fatigue	Severe leucopenia and plaquetopenia, respiratory distress, higher mortality	NS1 Antigen Test and IgM and IgG Antibody Tests	19, 23
West Nile virus	Asymptomatic, mild, neuroinvasive disease (rare)	Fatal neuroinvasive disease (often)	ELISA; RT-PCR	24,25
Zika	Fever, rash, joint pain, muscle pain, headache, conjunctivitis	Secondary bacterial infections and viral meningitis	Zika Virus RNA Testing (PCR) and IgM/IgG Serology	28,29
Yellow fever	Fever, headache, muscle and joint pain, fatigue	Liver failure, encephalopathy, and death	Yellow Fever Virus RNA Testing (PCR) and IgM/IgG Serology	31,32
Chikungunya	Fever, joint pain, muscle pain, rash, headache, fatigue	Milder symptoms	Chikungunya Virus RNA Testing (RT-PCR) and IgM/IgG Serology	36, 42
Hepatitis E	Jaundice, fatigue, abdominal pain, loss of appetite, nausea	Fulminant hepatitis, chronic hepatitis and cirrhosis	Hepatitis E RNA Detection (PCR) and IgM/IgG Serology	44, 51
Rabies virus	Fatal encephalitis	Fatal encephalitis	Microscopy; Direct fluorescent antibody; Immunohistochemistry; RT-PCR	53,54
SARS-CoV-2	Fever, cough, fatigue, vomiting, diarrhea	Severe outcome, respiratory insufficiency, and death	Antigen test and RT-PCR	55, 58
BKV polyomavirus	Asymptomatic	Ureteral stenosis and kidney dysfunction and inflammation	Urinary Polymerase Chain Reaction (PCR) and renal biopsy	77, 79
Cytomegalovirus	Fever, indisposition, loss of appetite	Increased incidence of morbidity and mortality	PCR, IgM/IgG Serology, ELISA method, Western Blot	80, 84

## Conclusion

The zoonotic origin of SARS-CoV-2 has raised more awareness of the possibility of new outbreaks and coinfection with potential zoonotic and organ failure-relevant viruses. Climate change, new ecological conditions, altered human behaviors, and increased human-animal interactions (pets and farming animals) are predisposing factors for disseminating viral diseases. In addition, increased urbanization and disruption of wildlife ecosystems bring more wild animals in close contact with the human vicinity, potentially increasing emerging viral exposure to human populations. In this review, we call for more awareness of the health impact of zoonotic viruses in SOT patients as a significant threat to global health and the need for permanent surveillance. More research is warranted to investigate their transmission dynamics pathophysiology in transplanted individuals to avoid organ rejection and mortality. These studies may also contribute to developing novel therapeutic strategies against emerging zoonotic viruses in crosstalk with other viruses inducing organ rejection.

In response to this challenge, vaccination, prophylaxis, and preemptive therapy are some strategies that have been developed and proven effective.

## Conflicts of interest

The authors declare no have conflicts of interest.
